# The impact of spatial arrangements on epidemic disease dynamics and intervention strategies

**DOI:** 10.1080/17513758.2016.1156172

**Published:** 2016

**Authors:** Michael R. Kelly, Joseph H. Tien, Marisa C. Eisenberg, Suzanne Lenhart

**Affiliations:** a Department of Mathematics, The Ohio State University, Columbus, OH, USA; b Departments of Epidemiology and Mathematics, University of Michigan, Ann Arbor, MI, USA; c Department of Mathematics, University of Tennessee, Knoxville, TN, USA

**Keywords:** Optimal control theory, ordinary differential equations, epidemiology

## Abstract

The role of spatial arrangements on the spread and management strategies of a cholera epidemic is investigated. We consider the effect of human and pathogen movement on optimal vaccination strategies. A metapopulation model is used, incorporating a susceptible–infected–recovered system of differential equations coupled with an equation modelling the concentration of *Vibrio cholerae* in an aquatic reservoir. The model compared spatial arrangements and varying scenarios to draw conclusions on how to effectively manage outbreaks. The work is motivated by the 2010 cholera outbreak in Haiti. Results give guidance for vaccination strategies in response to an outbreak.

## 1. Introduction

Questions have arisen regarding the importance of spatial features for disease spread and how movement patterns affect management strategies. Recently, population movement and water pathways impacted the spread of cholera through the departments of Haiti [13, 24, 31]. There is a need to find intervention strategies that could help control the disease while also optimizing the use of available resources. In addition, this strategy needs to consider the patterns of mobility of the population. The cholera situation in Haiti raised the interesting question: How do metapopulation spatial arrangements affect disease management strategy?

Cholera is an infectious disease caused by the infection of the intestine with the aquatic bacterium, *Vibrio cholerae*. In recent years, there have been several cholera epidemics throughout the world. The disease is also endemic in several areas of Asia and Africa. There are an estimated 3–5 million cholera cases and 100,000–120,000 deaths due to cholera every year [35]. The disease causes rapid dehydration and electrolyte imbalances. Without prompt treatment, a person with cholera may die of dehydration within a few hours of infection [11].

Cholera is a waterborne disease with multiple modes of infection, both direct/fast and indirect/slow transmission. Waterborne diseases are characterized by the capability for the disease to persist outside human hosts and transmission through aquatic sources is possible [35]. Cholera can be transmitted from an aquatic reservoir to human hosts through the ingestion of contaminated food or water. Infected individuals shed pathogen and new infections arise both from indirect exposure to contaminated water and from person-to-person transmission [29].

How the pathogen spreads to other communities can greatly affect disease dynamics. Previous work found evidence that the aquatic reservoir played an important role in the transmission of the disease [6, 7, 11, 31]. Pathogen movement through the population and environment have been suggested to influence the spatial spread of the disease in Haiti.

Tien and Earn [29] introduced a model using ordinary differential equations (ODEs) for multiple transmission pathways of a waterborne pathogen. They extended the traditional susceptible–infected–recovered compartmental model framework by adding a water compartment, tracking pathogen concentration in an aquatic reservoir. They introduced two modes of transmission, both direct and indirect transmission, allowing for infection through the environment as well as the classical person-to-person contact. Tuite *et al.* [31] applied the model to the 2010 cholera outbreak in Haiti. They investigated the spread of cholera in Haiti due to water contamination and human mobility by a gravity model, where the pathogen level was modelled as a function of both distance between communities as well as community size. Their model incorporated both fast and slow transmission but ignored water movement.

More recently, Eisenberg *et al.* [14] and Tien *et al.* [30] extended the model of Tien and Earn to incorporate a multi-patch structure with both pathogen and human movement. Global dynamics as determined by the domain ℛ_0_ are established in Eisenberg *et al.* Tien *et al.* give analytical formulas for the domain ℛ_0_. In particular, their results illustrate how network structure and patch characteristics combine to determine the ability of disease to invade the network. Tien *et al.* show that to the lowest order, the domain ℛ_0_ involves a weighted average of patch transmissibilities, with weights corresponding to the rooted spanning trees of the network (‘network risk’) [30]. This allows identification of communities that have the greatest contribution to the domain ℛ_0_.

More broadly, the use of multi-patch metapopulation models is an effective way to model the spread of a disease through a system of patches. There has been a large body of work in ecology using this modelling strategy [17, 22]. The use of metapopulation modelling has become popular in infectious disease modelling. One illustration is from Castillo-Chavez and Yakubu [8], who built an epidemic model for the dispersal of susceptible and infected individuals among patches in order to answer questions about the role of population dynamics on disease dynamics. Arino and his collaborators have also done work on epidemic models with spatial dynamics. Arino and van den Driessche [2] introduced a multi-city epidemic model that incorporated travel between the cities using directed graphs. Later work investigated multi-species models within a patchy environment with movement [4]. For a survey of other epidemic metapopulation models, see [1, 3, 19].

There are several intervention strategies to limit cholera outbreaks. Neilan *et al.* [23] used optimal control theory to study the effect of three control efforts on cholera outbreaks in a system of ODEs: vaccination, sanitation and the provision of clean water. Although improved water and sanitation infrastructure would be invaluable in preventing the spread of cholera, in many developing countries, this is not a viable short-term option. One short-term intervention strategy is the use of vaccinations. Vaccinations are currently being used as an intervention strategy to control cholera, specifically in Haiti [20]. In work by Chao *et al.* [9], an individual-based stochastic model for cholera transmission was analysed, comparing preemptive to responsive vaccination strategies.

*V. cholerae* has over 200 serotypes, however, only two cause epidemic disease, *V. cholerae* O1 and O139. There are currently two types of oral cholera vaccines available: Dukoral (WC-rBS) and the identical vaccines, Shanchol and mORCVAX. There are several differences between the two types. Dukoral consists of killed whole-cell *Vibrio cholerae* O1 while Shanchol (or mORCVAX) is a bivalent vaccine protecting against *V. cholerae* O1 and O139 [33]. Both are administered in two doses where Dukoral is administered for ages ≥ 2 at least a week apart, while Shanchol is for ages ≥ 1 administered 14 days apart. In addition, Dukoral requires a buffer or water for administration. Also, the cost of production of Shanchol is less than Dukoral, with a total cost of production and distribution ranging from $3 to 4 U.S. dollars per dose, depending on region demography [33].

Both vaccines offer significant protection against cholera during the first two years after vaccination. There have been significant studies in cholera vaccine efficacy for each type of vaccine in different areas of the world (see [10, 26, 28, 36]). The protective efficacy, found in these and other studies range from 86% to 66 % at 4–6 months, from 62% to 45 %, at one year, and from 77% to 58 % at 2 years after vaccination [33]. For more detailed information about oral cholera vaccines, see [27, 34].

To study the use of vaccinations to manage this disease effectively, we use optimal control together with models that incorporate varying spatial arrangements. We investigate how the spatial structure of patches with their corresponding connectivity can affect intervention strategies for disease outbreaks. We use an extension of the Tien and Earn model [29] to answer questions of where control efforts should be focused depending on spatial structure and patch dynamics. We start an investigation of certain spatial features and intervention strategies in epidemic models, motivated by the cholera outbreak in Haiti.

The model includes both population and pathogen movement. Incorporating water movement along hydrological connections is one way to model the spread of pathogen in aquatic reservoirs. By including both movement pathways, we decide how to apply control measures for a system of interconnected communities. We also investigate the importance of spatial connectivity of communities. We compare a linear spatial arrangement of connected patches with a hub arrangement with connecting smaller patches. The idea of a hub allows the model to account for patches of larger populations with higher connectivity to surrounding patches. The use of data [31] associated with the recent cholera outbreak in Haiti will help to analyze the effectiveness of our model. We apply a single control, vaccination, as an intervention and determine how the optimal control strategies in response to an outbreak depend on chosen spatial arrangements.

After a brief background on the Tien and Earn model [29], we introduce and describe our metapopulation model, as well as the parameters, for our problem in Section 2. In Section 3, we explain the role of connectivity and the associated spatial arrangements. The model is analysed by computing the basic reproduction number in Section 4. We formulate the optimal control problem and corresponding state system of equations in Section 5. We also prove the existence and uniqueness of the optimal control. In Section 6, we use numerical simulations to illustrate solutions to the problem for varying scenarios. The paper ends with a conclusion section.

## 2. Description of model

We extend the model of Tien and Earn [29] to include spatial movement among patches in a metapopulation setting. Each patch has dynamics modelled by a system of four ODEs (SIWR). The model incorporates both population movement and the hydrological links between communities that spread the disease. We also include a death due to disease term. The model accounts for both direct person-to-person transmission and indirect transmission from the environment to person. Infected individuals contribute the pathogen to an aquatic reservoir. The pathogen can then infect individuals within that community or could be transported to surrounding communities, infecting their aquatic reservoirs, which could lead to further transmission of the disease.

We investigate the cholera model with scaled pathogen concentration. The pathogen levels contributed to the water compartment from the infected population is known to be highly variable, depending on the individual. Due to the high variability in the ‘shedding’ rate, *α*, we scale the water compartment to eliminate the term from our model, using the scaling factor *ξ/αN*. We consider the system of equations:

(1) $$\frac{dS_{i}}{dt}=\mu_{i}N_{i}-\beta_{I}^{i}S_{i}I_{i}-\beta_{W}^{i}S_{i}W_{i}-\mu_{i}S_{i}+d_{S}\sum_{k=1}^{n}\left(M_{ik}S_{k}-M_{ki}S_{i}\right),$$

(2) $$\frac{dI_{i}}{dt}=\beta_{I}^{i}S_{i}I_{i}+\beta_{W}^{i}S_{i}W_{i}-(\gamma_{i}+\mu_{i}+\delta_{i})I_{i}+d_{I}\sum_{k=1}^{n}\left(M_{ik}I_{k}-M_{ki}I_{i}\right),$$

(3) $$\frac{dR_{i}}{dt}=\gamma_{i}I_{i}-\mu_{i}R_{i}+d_{R}\sum_{k=1}^{n}\left(M_{ik}R_{k}-M_{ki}R_{i}\right),$$

(4) $$\frac{dW_{i}}{dt}=\xi_{i}[I_{i}-W_{i}]+d_{W}\sum_{k=1}^{n}\left(H_{ik}W_{k}-H_{ki}W_{i}\right)-\phi_{i}W_{i},$$

subject to initial conditions:

(5) $$S_{i}(0)=S_{i_{0}},\;\;\;I_{i}(0)=I_{i_{0}},\;\;\;R_{i}(0)=R_{i_{0}},\;\;\;W_{i}(0)=W_{i_{0}}.$$

We let *M_i_*_,_*_j_* and *H_i_*_,_*_j_* be the connectivity of human and pathogen movement from patch *j* to patch *i*, respectively. We assume that the travel rate of individuals and pathogen from patch *j* to patch *i* is nonnegative, *M_i_*_,_*_j_* ≥ 0, *H_i_*_,_*_j_* ≥ 0, respectively. The parameters *μ* and *ξ* are positive, while the parameters *γ* and *δ* are nonnegative. We also assume that the transmission rates, *β_I_* and *β_W_*, are nonnegative. The compartments and parameters of the model with units are described in Tables 1 and 2, respectively.

Assume that the patches of the system are located along a river and that the patches are positioned such that one patch is located at the uppermost part of the river and another at the farthest downstream. Just as pathogen moves among the patches through the hydrological connections, there is a proportion of the pathogen that leaves the system at the bottom of the river and a smaller proportion leaving at the top. The patches located in the positions at the top and bottom of the river will be referred to as the source and terminal patches, respectively.

In order to appropriately model the pathogen movement in water through the metapopulation, we incorporate boundary conditions to account for pathogen leaving the system. We assume that the outbreak begins within the network; thus, there is no import of pathogen, after the initial time, from outside the system. The coefficient *ϕ_i_* appears in boundary patches, defined as follows:

(6) $$\phi_{i}=\left\{ \begin{array}{ll} \rho_{u} & \text{patch}\;i\;\text{is}\;\text{source},\\ \rho_{d} & \text{patch}\;i\;\text{is}\;\text{terminal},\\ 0 & \text{otherwise}, \end{array}\right.$$

where *ρ*_u_ and *ρ*_d_ are the coefficients for pathogen movement outside of the system in the upstream and downstream directions, respectively.

## 3. Role of connectivity and metapopulations

**Figure 1:**
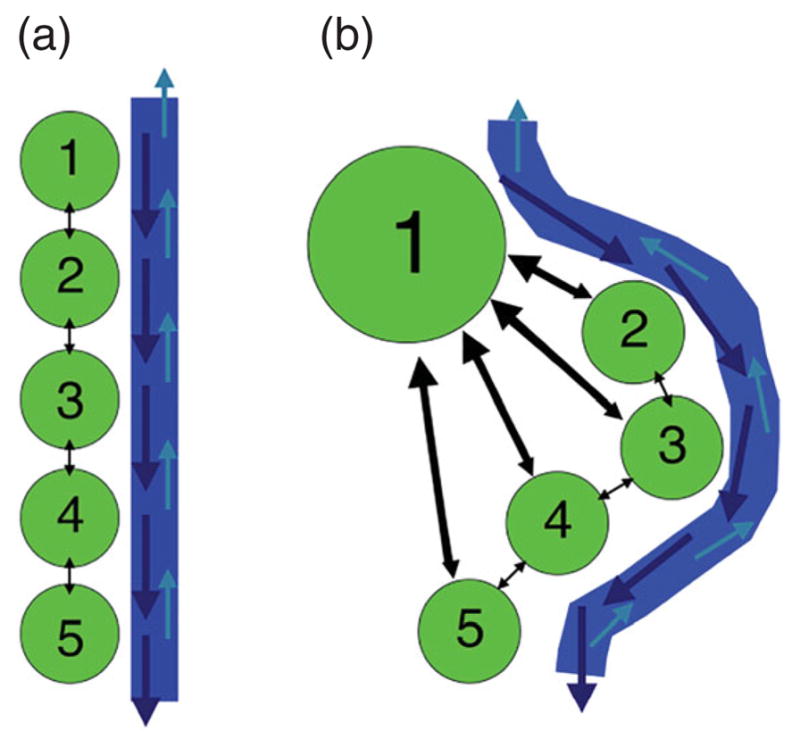
Visual description of five-patch linear (*left*) and hub (*right*) spatial arrangements.

We consider two types of spatial arrangements, each representing interconnected populations along with their corresponding water sources. The arrangements are weighted directional graphs involving both water and population movement. We specifically investigate spatial arrangements by varying their connectivities, rates of movement, parameters, and population sizes. The connectivity patterns of linear and hub arrangements can be visually represented in Figure 1.

### 3.1. Linear spatial arrangement

Our first scenario is a sequence of patches along a river, where all travel is between nearest neighbours only. The connectivity matrix for population movement in the *n*-patch linear spatial arrangement is given by

(7) $$M=\left[\begin{array}{ccccc} 0 & M_{12} & 0 & \ldots & 0\\ M_{21} & 0 & M_{23} & \ddots & \vdots \\ 0 & M_{32} & 0 & \ddots & 0\\ \vdots & \ddots & \ddots & \ddots & M_{n-1,n}\\ 0 & \ldots & 0 & M_{n,n-1} & 0 \end{array}\right].$$

We assume that individuals move bidirectionally, moving both to and from a patch, at identical rates, thus the coefficients are equal in both directions, *M_i_*_,_*_j_* = *M_j_*_,_*_i_* for all *i,j*.

The connectivity matrix for water movement has an identical form:

(8) $$H=\left[\begin{array}{ccccc} 0 & H_{12} & 0 & \ldots & 0\\ H_{21} & 0 & H_{23} & \ddots & \vdots \\ 0 & H_{32} & 0 & \ddots & 0\\ \vdots & \ddots & \ddots & \ddots & H_{n-1,n}\\ 0 & \ldots & 0 & H_{n,n-1} & 0 \end{array}\right].$$

We assume that pathogen moves in both the up and downstream directions, however, due to the directional flow of the river, the movement rate downstream will be larger than upstream. This natural occurrence is incorporated into the model through the coefficients of the connectivity matrix. Thus, *H_i_*_,_*_j_* (downstream) *> H_j_*_,_*_i_* (upstream) for *i > j*.

### 3.2. Hub spatial arrangement

We compare the linear spatial arrangement with a scenario which incorporates a hub patch, representing a large, populous area with higher human movement to and from each of the other patches in the system. For example, assuming Patch 1 is the hub, the coefficients *M*_1_*_j_* and *M_i_*_1_ for *i*, *j* = 1, …, *n*, represent movement to and from the hub patch, with the following inequality conditions, *M*_1_*_j_ > M_i_*_,_*_j_* for all *i*, *j* ≠1.

We still have the patches located along a linear river arrangement and assume movement of the population in non-hub patches is to the hub and its nearest neighbours. The connectivity matrix for population movement in an *n*-patch hub arrangement, with Patch 1 as hub is given by

(9) $$M=\left[\begin{array}{cccccc} 0 & M_{12} & M_{13} & \ldots & \ldots & M_{1,n}\\ M_{21} & 0 & M_{23} & 0 & \ldots & 0\\ M_{31} & M_{32} & \ddots & \ddots & \ddots & \vdots \\ \vdots & 0 & \ddots & \ddots & \ddots & 0\\ \vdots & \vdots & \ddots & \ddots & \ddots & M_{n-1,n}\\ M_{n,1} & 0 & \ldots & 0 & M_{n,n-1} & 0 \end{array}\right].$$

We consider the same pattern of pathogen movement in water as the linear spatial arrangement, thus the same connectivity matrix (Equation (8)).

## 4. Basic reproduction number

We begin by finding the basic reproduction number for our model. We use the next-generation approach, introduced by Diekmann and Heesterbeek [12] and Watmough and van den Driessche [32]. We arrange our system of equations into the form *x̄′*= ℱ − 𝒱, with ℱ containing new infected terms and 𝒱 containing transition terms, with the order of components being *(I*_1_, …, *I_n_*, *W*_1_, …, *W_n_*, *S*_1_, …, *S_n_*, *R*_1_, …, *R_n_)*. The equations of our system that correspond to infected compartments are the following. For *i* = 1, …, *n*

$${\left[\begin{array}{c} I_{i}\\ W_{i} \end{array}\right]}^{\prime }=\left[\begin{array}{c} \beta_{I}^{i}S_{i}I_{i}+\beta_{W}^{i}S_{i}W_{i}\\ 0 \end{array}\right]-\left[\begin{array}{c} (\gamma_{i}+\mu_{i}+\delta_{i})I_{i}-d_{I}\sum_{k=1}^{n}\left[M_{ik}I_{k}-M_{ki}I_{i}\right]\\ -\xi_{i}(I_{i}-W_{i})-d_{W}\sum_{k=1}^{n}\left[H_{ik}W_{k}-H_{ki}W_{i}\right]+\phi_{i}W_{i} \end{array}\right].$$

A unique disease-free equilibrium (DFE) exists since removing the infected population and pathogen leaves a fully susceptible population that will remain disease free. The DFE is found by setting *I_i_* = *W_i_* = 0 for *i* = 1, …, *n*, and calculating the null vector of the Laplacian of the human movement matrix, *M*:

$$L_{M}=\left[\begin{array}{cccc} d_{S}\sum_{k=1}^{n}M_{ki} & -d_{S}M_{12} & \ldots & -d_{S}M_{1n}\\ -d_{S}M_{21} & \ddots & \ddots & \vdots \\ \vdots & \ddots & \ddots & -d_{S}M_{n-1n}\\ -d_{S}M_{n1} & \ldots & -d_{S}M_{nm-1} & d_{S}\sum_{k=1}^{n}M_{ki} \end{array}\right].$$

The DFE can be written as

$$\text{DFE}=(S_{1},\ldots ,S_{n},I_{1},\ldots ,I_{n},R_{1},\ldots ,R_{n},W_{1},\ldots ,W_{n})=(S_{1}^{*},\ldots ,S_{n}^{*},0,\ldots ,0),$$

where $S_{i}^{*}=(\sum_{i=1}^{n}N_{i})u_{i}$, and *u_i_*, referred to as the network risk [30], is the component of the null vector such that $\sum_{i=1}^{n}u_{i}=1$. By taking the Jacobian of the submatrix of ℱ with respect to each of the infected and water compartments and then evaluating at the DFE, we get the following 2*n* × 2*n* matrix, *F*:

$$F=\left[\begin{array}{cc} F_{11} & F_{12}\\ 0 & 0 \end{array}\right],$$

where $F_{11}=\text{diag}(\beta_{I}^{i}S_{i}^{*})$ and $F_{12}=\text{diag}(\beta_{W}^{i}S_{i}^{*})$, both *n* × *n* matrices. Similarly, taking the Jacobian of the submatrix of 𝒱 with respect to the infected and water compartments and evaluating at the DFE, we get the 2*n* × 2*n* matrix, *V*:

$$V=\left[\begin{array}{cc} V_{11} & 0\\ -V_{21} & V_{22} \end{array}\right],$$

where *V*_21_ = diag*(ξ_i_)*, an *n* × *n* matrix,

$$\begin{array}{l} V_{11}=\left[\begin{array}{cccc} A_{1} & -d_{I}M_{12} & \ldots & -d_{I}M_{1n}\\ -d_{I}M_{21} & \ddots & \ddots & \vdots \\ \vdots & \ddots & \ddots & -d_{I}M_{n-1n}\\ -d_{I}M_{n1} & \ldots & -d_{I}M_{nm-1} & A_{n} \end{array}\right],\\ V_{22}=\left[\begin{array}{cccc} B_{1} & -d_{W}H_{12} & \ldots & -d_{W}H_{1n}\\ -d_{W}H_{21} & \ddots & \ddots & \vdots \\ \vdots & \ddots & \ddots & -d_{W}H_{n-1n}\\ -d_{W}H_{n1} & \ldots & -d_{W}H_{nm-1} & B_{n} \end{array}\right], \end{array}$$

and

$$\begin{array}{ll} A_{i}\; & =(\gamma_{i}+\mu_{i}+\delta_{i})+d_{I}\sum_{k=1}^{n}M_{ki},\\ B_{i}\; & =\xi_{i}+d_{W}\sum_{k=1}^{n}H_{ki}+\phi_{i}. \end{array}$$

We use an approach by Arino [1], incorporating block matrices to compute the basic reproduction number. The matrices *V*_11_ and *V*_22_ are both non-positive on the off-diagonal and satisfy the definition of non-singular M-Matrices, meaning that they can be written in the form, *A* = *sI*−*B*, where *s >* 0 and *B* ≥ 0, and *s > ρ(B)*, the spectral radius of *B*. Since both matrices, *V*_11_ and *V*_22_, are non-singular M-Matrices, they have positive inverses and are both irreducible [5]. Thus, we have

$$V^{-1}=\left[\begin{array}{cc} V_{11}^{-1} & 0\\ V_{22}^{-1}V_{21}V_{11}^{-1} & V_{22}^{-1} \end{array}\right].$$

We form the next-generation matrix, *FV*^−1^:

$$FV^{-1}=\left[\begin{array}{cc} F_{11}V_{11}^{-1}+F_{12}V_{22}^{-1}V_{21}V_{11}^{-1} & F_{12}V_{22}^{-1}\\ 0 & 0 \end{array}\right].$$

The spectral radius for *FV*^−1^ is the same as the spectral radius of the matrix in the upper left block of *FV*^−1^,

$$R_{0}=\rho (FV^{-1})=\rho (F_{11}V_{11}^{-1}+F_{12}V_{22}^{-1}V_{21}V_{11}^{-1}),$$

which is dependent on spatial structure and the corresponding connectivity matrices, *M* and *H*. In the case of a single patch, ℛ_0_ simplifies to the following:

$$R_{0}=\frac{(\beta_{I}+\beta_{W})N}{\gamma +\mu +\delta }.$$

From [32], we obtain the stability result: When ℛ_0_ *<* 1, the disease-free equilibrium is locally asymptotically stable but if ℛ_0_ *>* 1, the equilibrium will be unstable.

## 5. Optimal control formulation and analysis

To investigate how the control strategy depends on the spatial arrangement of patches, we include vaccination in the system (1)–(4). The term, v*_i_(t)*, represents the rate of vaccine effort transferring susceptibles directly to the recovered class within patch *i*. Those in the recovered class are now considered immune or ‘removed.’ The term, v*_i_(t)* = *ησ_i_(t)*, is a combined coefficient where *η* represents the efficacy of the vaccination distribution and *σ_i_* is the rate of vaccine distribution. We assume that individuals removed from the susceptible class have received the complete two-dose vaccine program. When including vaccination terms, v*_i_(t)*, our metapopulation takes the form:

(10) $$\frac{dS_{i}}{dt}=\mu_{i}N_{i}-\beta_{I}^{i}S_{i}I_{i}-\beta_{W}^{i}S_{i}W_{i}-\mu_{i}S_{i}+d_{S}\sum_{k=1}^{n}\left(M_{ik}S_{k}-M_{ki}S_{i}\right)-v_{i}(t)S_{i},$$

(11) $$\frac{dI_{i}}{dt}=\beta_{I}^{i}S_{i}I_{i}+\beta_{W}^{i}S_{i}W_{i}-(\gamma_{i}+\mu_{i}+\delta_{i})I_{i}+d_{I}\sum_{k=1}^{n}\left(M_{ik}I_{k}-M_{ki}I_{i}\right),$$

(12) $$\frac{dR_{i}}{dt}=\gamma_{i}I_{i}-\mu_{i}R_{i}+d_{R}\sum_{k=1}^{n}\left(M_{ik}R_{k}-M_{ki}R_{i}\right)+v_{i}(t)S_{i},$$

(13) $$\frac{dW_{i}}{dt}=\xi_{i}[I_{i}-W_{i}]+d_{W}\sum_{k=1}^{n}\left(H_{ik}W_{k}-H_{ki}W_{i}\right)-\phi_{i}W_{i},$$

with previous initial conditions (5).

We seek to find an optimal vaccination strategy that minimizes the number of infecteds in the network while minimizing some nonlinear cost involved with the vaccination program. The cost involved with the vaccination strategy includes two terms. We have a nonlinear cost associated with vaccination which incorporates the cost of housing distribution centres, employing individuals to administer the vaccines, and other large costs for implementing a vaccination campaign. The linear part represents the total number of susceptibles vaccinated, which includes the cost of vaccination for each individual. See [16] for some justification for including such a nonlinearity in the cost with vaccination control.

The objective functional is given by

(14) $$J(v)=\int_{0}^{T}\sum_{i=1}^{n}A_{i}I_{i}+\sum_{i=1}^{n}B_{i}v_{i}^{2}+\sum_{i=1}^{n}C_{i}v_{i}S_{i}\;dv$$

over the control set:

$$V=\{v=(v_{1},\ldots ,v_{n})|v_{i}:[0,T]\to \mathbb{R},0\leq v_{i}(t)\}\leq v_{\max},v_{i}\;\text{Lebesguemeasurable}\}.$$

The positive constants, *A_i_*, *B_i_*, *C_i_*, are used to weight the relative importance of each of the terms in the objective functional. The optimal control problem is stated as follows.

Find v^*^ ∈ *V*

$$J(v^{*})=\underset{v\in V}{\inf}J(v)$$

such that subject to state equations (10)–(13) and initial conditions (5).

Now that we have formulated the optimal control problem, we can show easily that there exists a nonnegative, bounded solution to the initial value problem defined by Equations (10)–(13) with initial conditions (5). By a similar approach as [30] and using assumptions on the model, we see the system is positively invariant. Thus, the solutions of the system, starting with nonnegative initial conditions, stay nonnegative for all *t >* 0. In proving the boundedness of the state system solutions, we follow a similar approach as [14].

### 5.1. Existence of the optimal control

In order to use Pontryagin’s maximum principle (PMP) [25], the existence of an optimal control must be proven.

#### Theorem 5.1

*There exists an optimal control vector,* $v^{*}=(v_{1}^{*},\ldots ,v_{n}^{*})\in V$ *with corresponding states* $x^{*}=(S_{1}^{*},I_{1}^{*},R_{1}^{*},W_{1}^{*},\ldots ,S_{n}^{*},I_{n}^{*},R_{n}^{*},W_{n}^{*})$, *that minimizes the objective functional J(*v*) defined by *Equation (14).

##### Proof

Since objective functional values are nonnegative, there exists a minimizing sequence of controls, $v^{k}=(v_{1}^{k},\ldots ,v_{n}^{k})$, in *V* such that

$$\lim_{k\to \infty }J(v^{k})=\underset{v\in V}{\inf}J(v).$$

The controls in *V* are uniformly bounded in *L*^∞^ and by Friedman [15], there exists v^*^ ∈ *V* such that on a subsequence, for each *i*,

$$v_{i}^{k}⇀v_{i}^{*}\;\text{weakly}\;\text{in}\;L^{2}([0,T])\;\text{as}\;k\to \infty .$$

To pass to the limit in the ODEs, we need a stronger convergence on the states. The state sequence corresponding to the sequence of minimizing controls is also uniformly bounded. Then, the right-hand sides of the ODE system are uniformly bounded and we obtain uniformly bounded derivatives, for all *k* and *i* = 1, …, *n*. Thus, the state solution sequence {**x***^k^*} is equicontinuous, and by the Arzelà–Ascoli Theorem, there exists x^*^ such that

$$x^{k}\to x^{*}\;\text{uniformly}\;\text{on}\;[0,T].$$

This uniform convergence is needed to get convergence of terms like v*_i_S_i_* in our system. By passing the limit in the system of differential equations, we can show that x^*^ is the state corresponding to the control v^*^. Using lower semicontinuity of *L*^2^ norms with respect to *L*^2^ weak convergence and the convergences above, we have

$$\underset{v}{\inf}J(v)=\lim_{k\to \infty }J(v^{k})\geq \int_{0}^{T}\sum_{i=1}^{n}(A_{i}I_{i}^{*}+C_{i}v_{i}^{*}S_{i}^{*})\;dv+\int_{0}^{T}\sum_{i=1}^{n}B_{i}{\left(v_{i}^{*}\right)}^{2}dv=J(v^{*}).$$

Thus, v^*^ is an optimal control.

### 5.2. Optimality system

Having obtained the existence of an optimal control, we can now apply PMP, forming the Hamiltonian, **H**:

(15) $$\begin{array}{l} H=\sum_{i=1}^{n}\left[A_{i}I_{i}+B_{i}v_{i}^{2}+C_{i}v_{i}S_{i}\right]\\ +\sum_{i=1}^{n}\lambda_{S_{i}}\;\left[\mu_{i}N_{i}-\beta_{I}^{i}S_{i}I_{i}-\beta_{W}^{i}S_{i}w_{i}-\mu_{i}S_{i}+d_{S}\sum_{k=1}^{n}\left(M_{ik}S_{k}-M_{ki}S_{i}\right)-v_{i}(t)S_{i}\right]\\ +\sum_{i=1}^{n}\lambda_{I_{i}}\;\left[\beta_{I}^{i}S_{i}I_{i}+\beta_{W}^{i}S_{i}w_{i}-(\gamma_{i}+\mu_{i}+\delta_{i})I_{i}+d_{I}\sum_{k=1}^{n}\left(M_{ik}I_{k}-M_{ki}I_{i}\right)\right]\\ +\sum_{i=1}^{n}\lambda_{R_{i}}\;\left[\gamma_{i}I_{i}-\mu R_{i}+d_{R}\sum_{k=1}^{n}\left(M_{ik}R_{k}-M_{ki}R_{i}\right)+v_{i}(t)S_{i}\right]\\ +\sum_{i=1}^{n}\lambda_{w_{i}}\;\left[\xi_{i}(I_{i}-w_{i})+d_{W}\sum_{k=1}^{n}\left(H_{ik}w_{k}-H_{ki}w_{i}\right)-\phi_{i}w_{i}\right], \end{array}$$

where the adjoint variables (*λ_j_*), corresponding to their respective states, attach the right-hand side of the state equations to the objective functional. Since an optimal solution exists, we can obtain the necessary conditions for optimality using Pontryagin’s maximum principle.

#### Theorem 5.2

*Given an optimal control vector* v^*^ ∈ *V*, *and corresponding states* x^*^, *there exist adjoint functions satisfying*

(16) $$\frac{d\lambda_{S_{i}}}{dt}=-\left(C_{i}v_{i}(t)+\lambda_{S_{i}}\;\left[-\beta_{I}^{i}I_{i}-\beta_{W}^{i}w_{i}-d_{S}\sum_{k=1}^{n}M_{ki}-v_{i}(t)\right]+\lambda_{I_{i}}[\beta_{I}^{i}I_{i}+\beta_{W}^{i}w_{i}]+\lambda_{R_{i}}v_{i}(t)+d_{S}\sum_{k=1}^{n}\left(\lambda_{S_{k}}M_{ki}\right)\right),$$

(17) $$\frac{d\lambda_{I_{i}}}{dt}=-\left(A_{i}+\lambda_{S_{i}}(\mu_{i}-\beta_{I}^{i}S_{i})+\lambda_{I_{i}}\;\left[\beta_{I}^{i}S_{i}-(\gamma_{i}+\mu_{i}+\delta_{i})-d_{I}\sum_{k=1}^{n}M_{ki}\right]+\lambda_{R_{i}}\gamma_{i}+\lambda_{W_{i}}\xi_{i}+d_{I}\sum_{k=1}^{n}\left(\lambda_{I_{k}}M_{ki}\right)\right),$$

(18) $$\frac{d\lambda_{R_{i}}}{dt}=-\left(\lambda_{S_{i}}\mu_{i}-\lambda_{R_{i}}(\mu_{i}+d_{R}\sum_{k=1}^{n}M_{ki})+d_{R}\sum_{k=1}^{n}\left(\lambda_{R_{k}}M_{ki}\right)\right),$$

(19) $$\frac{d\lambda_{W_{i}}}{dt}=-\left(-\lambda_{S_{i}}\beta_{W}^{i}S_{i}+\lambda_{I_{i}}\beta_{W}^{i}S_{i}-\lambda_{W_{i}}\;\left(\xi_{i}+d_{W}\sum_{k=1}^{n}H_{ki}+\phi_{i}\right)+d_{W}\sum_{k=1}^{n}\left(\lambda_{W_{k}}H_{ki}\right)\right),$$

*for i* = 1, …, *n with the following transversality conditions at the final time, T,*

(20) $$\lambda_{S_{i}}(T)=\lambda_{I_{i}}(T)=\lambda_{R_{i}}(T)=\lambda_{W_{i}}(T)=0.$$

This optimal control is characterized by

(21) $$v_{i}^{*}=\max\;\left(\min\;\left(v_{\max},\frac{\lambda_{S_{i}}S_{i}-\lambda_{R_{i}}S_{i}-C_{i}S_{i}}{2B_{i}}\right),0\right).$$

##### Proof

The differential equations for the adjoint are standard results of Pontryagin’s maximum principle [25]. The right-hand sides of the differential equations are easily computed by

$$\frac{d\lambda_{S_{i}}}{dt}=-\frac{\partial H}{\partial S_{i}},\;\;\;\frac{d\lambda_{I_{i}}}{dt}=-\frac{\partial H}{\partial I_{i}},\;\;\;\frac{d\lambda_{R_{i}}}{dt}=-\frac{\partial H}{\partial R_{i}},\;\;\;\frac{d\lambda_{W_{i}}}{dt}=-\frac{\partial H}{\partial W_{i}}.$$

The final time conditions are the transversality conditions. The adjoint differential equations are linear in the adjoint function, and thus a unique adjoint solution exists.

Using standard arguments from ∂**H***/*∂*v_i_*, we have a characterization for this optimal control (Equation (21)). Also note, $\partial^{2}H/\partial v_{i}^{2}=2B_{i}>0$ confirming that our optimal control minimizes the Hamiltonian.

The optimality system for our problem consists of the state equations (10)–(13), the adjoint equations (16)–(19), and the control characterization (Equation (21)).

## 6. Numerical simulations

The stability and control analysis were completed for any finite number of patches. For numerical purposes, we consider a five patch network of varying connectivities. We will consider linear and hub arrangements. Initially, we assume identical patch dynamics. We later investigate the role of ‘hot spots’ (HSs), or patches with higher infectivity.

The forward–backward iterative technique [21] was used to solve the optimality system, which consists of Equations (10)–(13), (16)–(19), and (21). To solve the ODE systems, we use a fourth-order Runge–Kutta method.

### 6.1. Linear spatial arrangement

We first investigate disease dynamics and optimal vaccination strategies within a system of patches with a linear spatial arrangement, illustrated in Figure 1 and described in Section 3.1. The parameter values chosen are found in Table 3. Parameter values were chosen from the work of Tuite *et al.* [31] based on the outbreak in Haiti. The maximum vaccination rate was chosen to resemble the Partners in Health pilot vaccination campaign in Bocozelle, Haiti [18]. The model is subject to initial conditions that depend on the outbreak patch. The weight coefficients in *J* were chosen to represent approximate costs of implementing a vaccination program in Haiti.

We assume that the values of the non-zero elements of the movement adjacency matrix, *M_i_*_,_*_j_*, are identical and equal to one. The value for the non-zero downstream connectivity movements, *H_i_*_,_*_j_* where *i > j*, is equal to one, while for the non-zero upstream connectivity movements, *H_i_*_,_*_j_* for *j > i*, was chosen to be one-tenth the rate of the downstream movement. This is due to a stronger movement of pathogen downstream than upstream, which is consistent with the direction of river flow.

For all simulations, the outbreak patch begins with 9700 susceptible individuals and 300 infected. This was chosen to simulate an epidemic, where a significant number of infected would need to be present in a community before a large-scale intervention strategy would be implemented. We assume the same value of 300 for the scaled pathogen concentration, *W*_0_, to match the number of infecteds in the outbreak patch. To ensure numerical convergence of the optimal control, we require trace amounts of pathogen to be present in all patches. This may reflect a more biologically realistic scenario, as an epidemic with 300 individuals would likely have spread at least small amounts of pathogen to the other water compartments. We took the non-outbreak patches to have small, decreasing levels of pathogen as we move away from the outbreak patch, that is, for an outbreak in Patch 1, *W*_0_ = [300, 1, 0.5, 0.1, 0.05]. We assume that each non-outbreak patch begins with 10,000 susceptibles so the metapopulation totals 50,000 individuals. We focus our study on outbreaks occurring either at the head of the river network or centralized in the metapopulation, that is, in Patch 1 and Patch 3, respectively. We also discuss outbreaks at the lower end of the river, in Patch 5.

**Figure 2:**
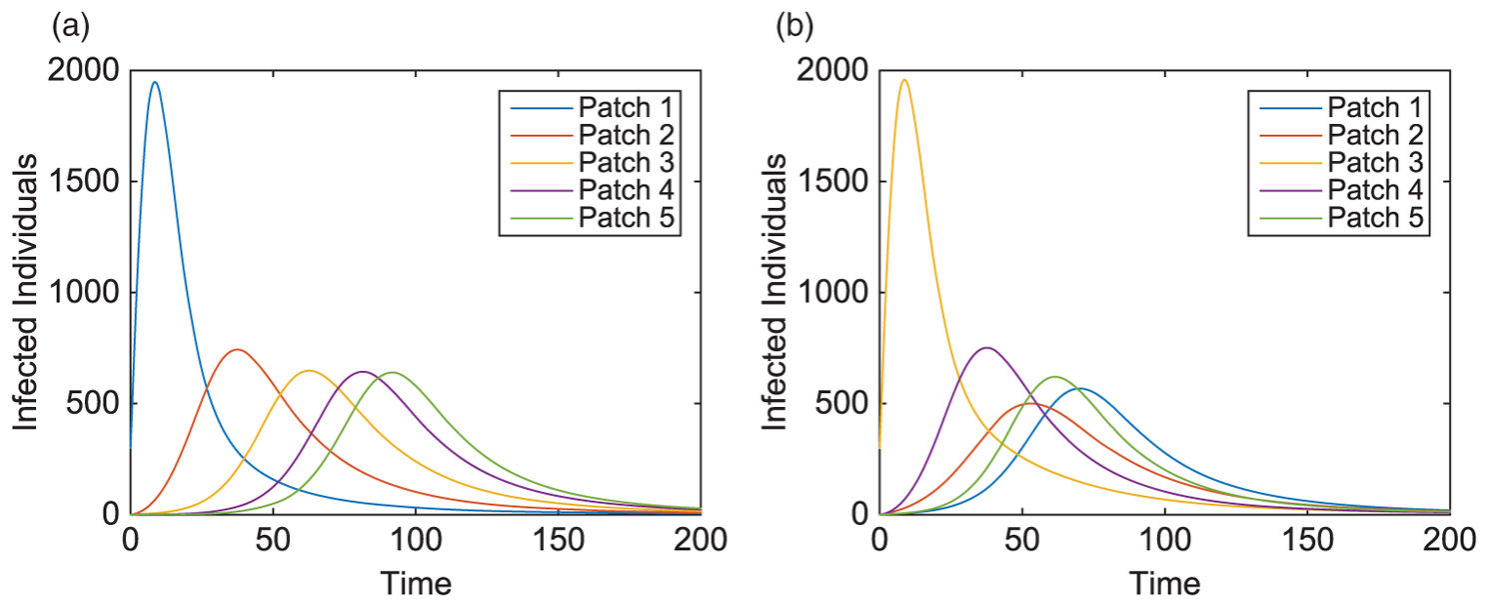
Linear arrangement: infected population dynamics when outbreak occurs upstream and centre of metapopulation (without vaccination).

Without vaccination, the dynamics of the infected population for two outbreak scenarios is given in Figure 2. Notice the patterns that form as the disease travels to the nearest neighbour throughout the metapopulation. We also see the disease spreading to patches downstream at a faster rate than those upstream. Due to each patch having identical dynamics, the basic reproduction number for each patch is $R_{0}^{i}=5.88$ for *i* = 1, …, 5, while the basic reproduction number of the network is

$$R_{0}=5.04.$$

The difference in the domain **R**_0_ and the patch $R_{0}^{i}$ values is due to the boundary conditions applied to the source and terminal patches removing pathogen from the system.

**Figure 3:**
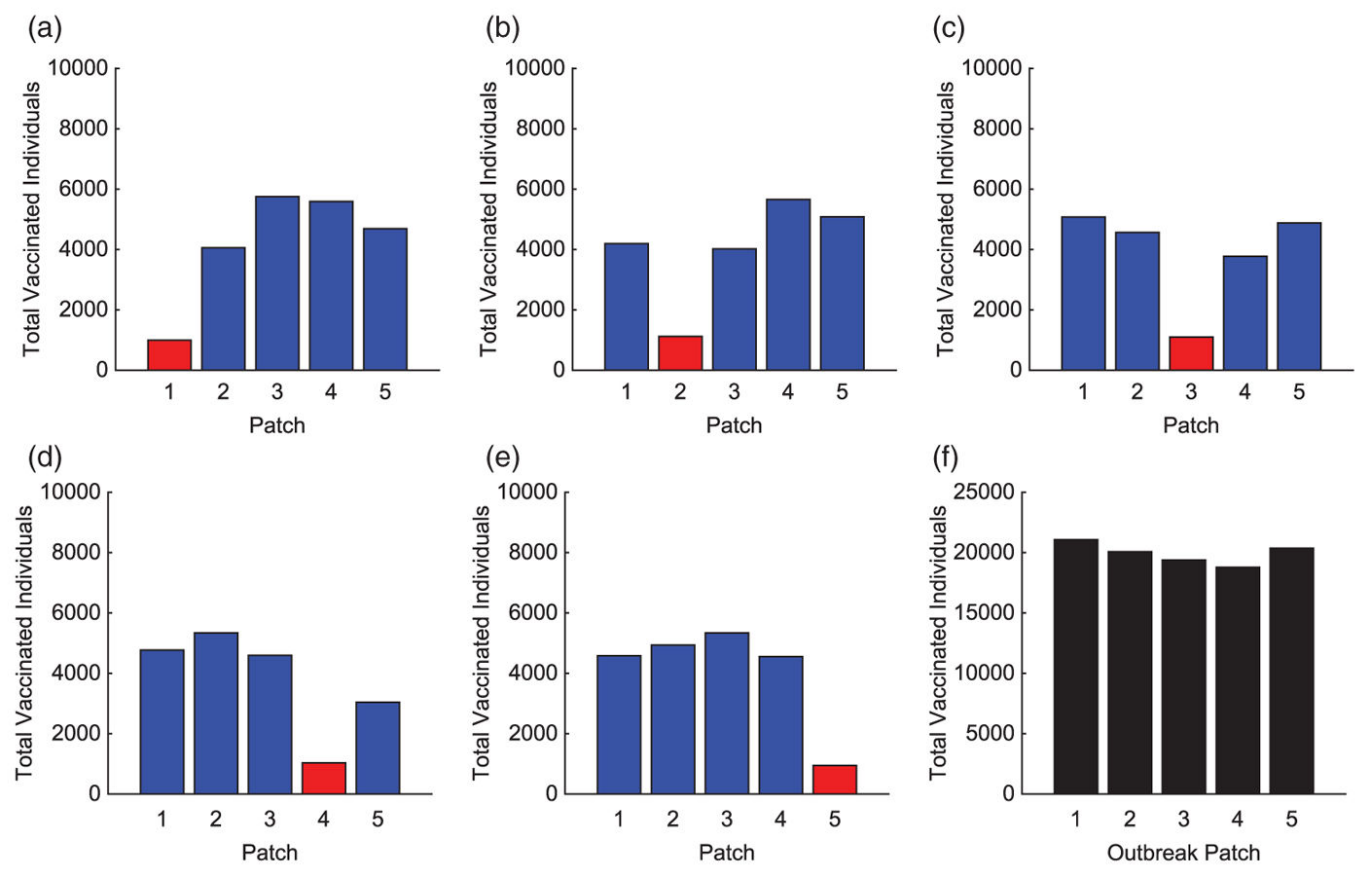
Linear arrangement: total individuals vaccinated in each patch for each scenario. The total vaccinated in metapopulation for each scenario is given in (f). (The outbreak patch is shown in red.)

**Figure 4:**
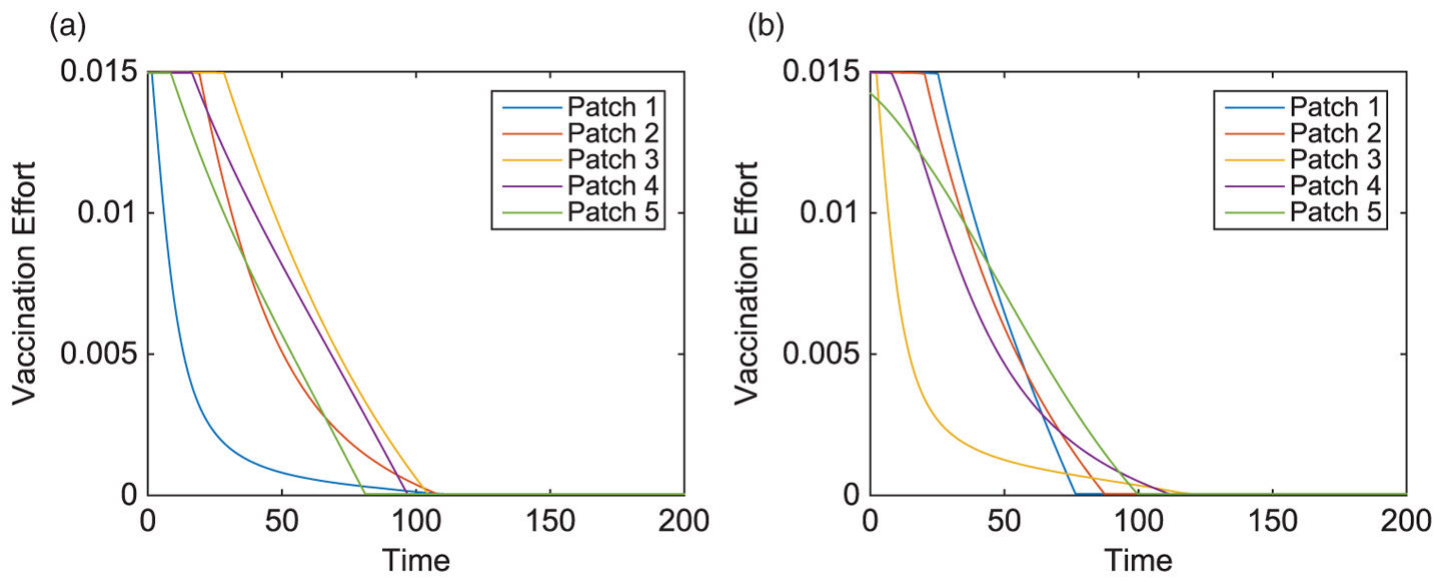
Linear arrangement: vaccination effort of patches with outbreaks in Patch 1 and Patch 3.

**Figure 5:**
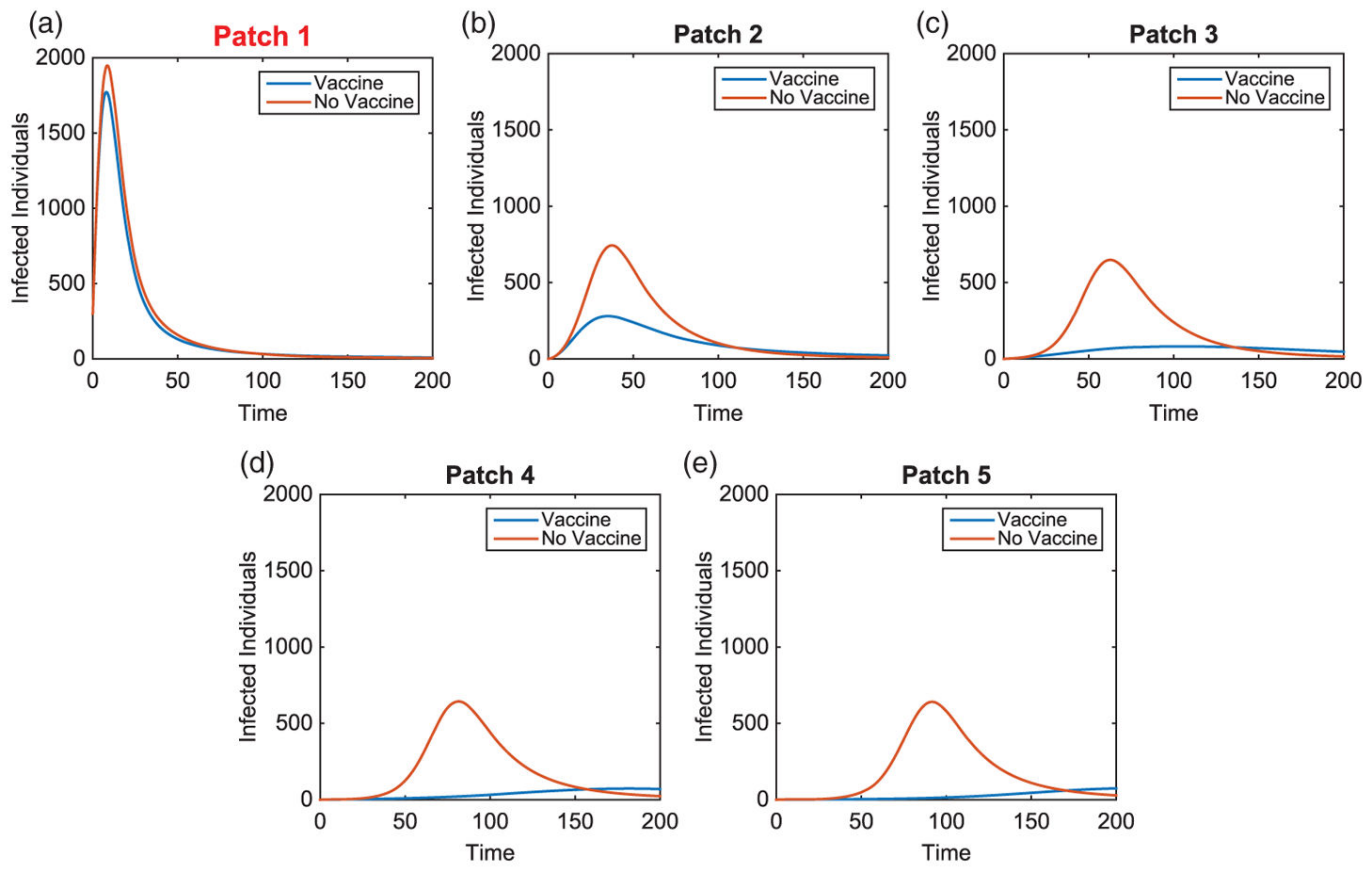
Linear arrangement: infected population dynamics comparison with and without vaccination with outbreak in Patch 1.

**Figure 6:**
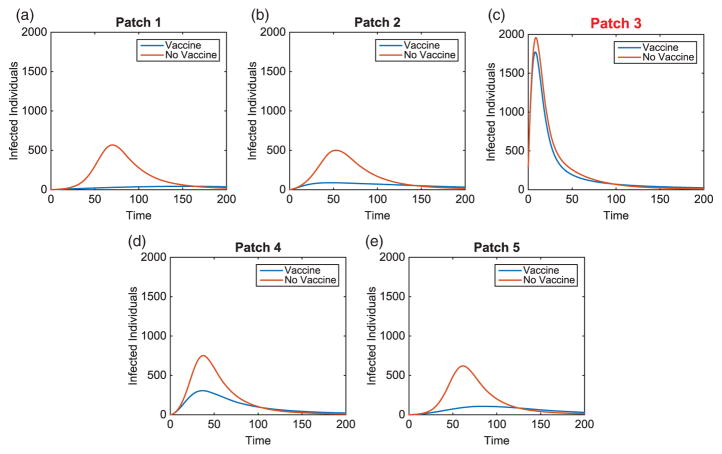
Linear arrangement: infected population dynamics comparison with and without vaccination with outbreak in Patch 3.

We consider specific linear arrangement scenarios to illustrate the dependence of the intervention strategy on outbreak location. For each scenario, the total infected with and without vaccination is listed in Table 4. The total vaccinated in each patch of the metapopulation for each outbreak case is illustrated in Figure 3. We illustrate our results for varying outbreak scenarios in Figures 4–6. For convenience, we refer to the vaccination effort as the control level and vaccination rate as the number of individuals vaccinated per time. We report the control effort, the total number of susceptible individuals vaccinated in each patch at each time, and how the control strategy affected the infected population.

For linear arrangements, we can draw several conclusions. It is evident that the optimal control strategies depend on where outbreaks occur. Effort is always focused on patches neighbouring the outbreak patch, whether a direct or close proximity neighbour (see Figure 3).

Outbreaks upstream result in higher control efforts in near-neighbouring patches downstream to prevent disease invasion at the bottom of the network. The vaccination strategy shifts with outbreaks occurring lower in the metapopulation. Due to more susceptible patches upstream and the slower rate of pathogen movement upstream than down, more effort is spent protecting patches immediately upstream.

In all cases, we saw significant decreases in the number of infecteds in all patches outside of the outbreak patch. This is due to the difficulty in controlling a patch by vaccination that is already invaded by the disease. Although the total distribution of vaccination is almost uniform for each of the outbreak scenarios (see Figure 3(f)), the effects are quite different. The vaccination strategy has the greatest effect in lowering the number of infected individuals when the outbreak occurs downstream in the river network. It is evident that outbreaks occurring in a central location, especially if farther upstream compared to closer to the bottom of the patch network, are more complicated to control effectively, as seen in Table 4.

### 6.2. Hub spatial arrangements

We now introduce patches known as hubs, as discussed in Section 3.2. We consider a five-patch hub spatial arrangement illustrated in Figure 1, although we vary the location of the hub patch. We consider scenarios when an outbreak occurs in the hub or in surrounding patches. We consider an initial population at equilibrium, with a hub size of 18,000 individuals and 8000 individuals in surrounding patches. In all cases, we still consider 300 infected individuals and either 17,700 or 7700 susceptibles, respectively, wherever the outbreak occurs. The same initial conditions as the linear arrangement are used for the water compartment. We assume that all patches outside the hub are identical (apart from their initial conditions).

The basic reproduction numbers of the hub (regardless of location) and surrounding patches are

(22) $$R_{0}^{i}=\left\{ \begin{array}{ll} 10.59 & \text{patch}\;i\;\text{is}\;\text{hub},\\ 4.71 & \text{otherwise.} \end{array}\right.$$

The basic reproduction number of the network is determined by the network structure and thus depends on hub patch location. The values for three distinct hub locations in network are recorded in Table 5. The reproduction number is independent of where the outbreak begins.

**Figure 7:**
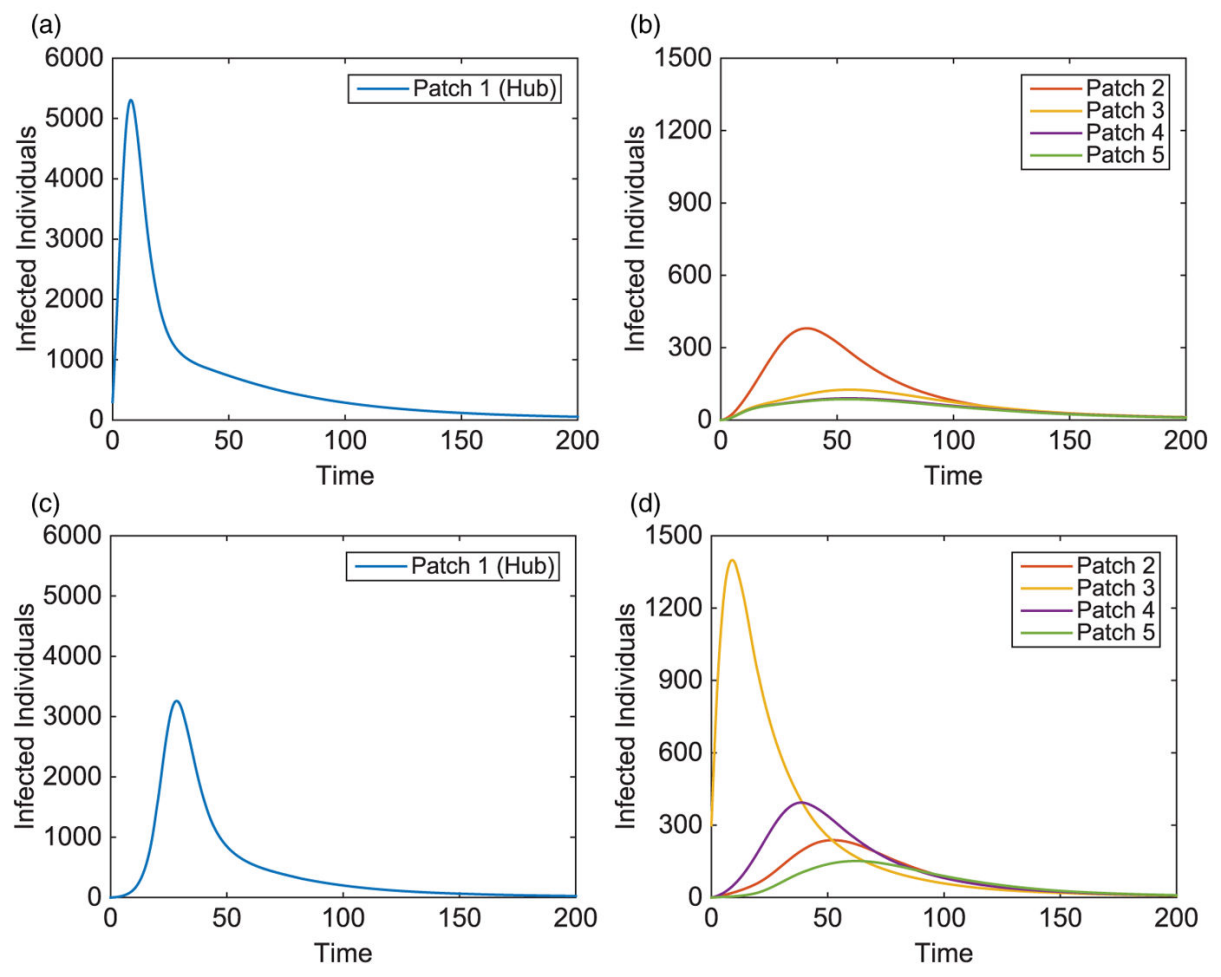
Hub Patch 1 arrangement: infected population dynamics when outbreak occurs in two distinct areas, Patches 1 and 3, of the metapopulation (without vaccination), where plot on the left is the hub only, plot on the right is surrounding patches.

**Figure 8:**
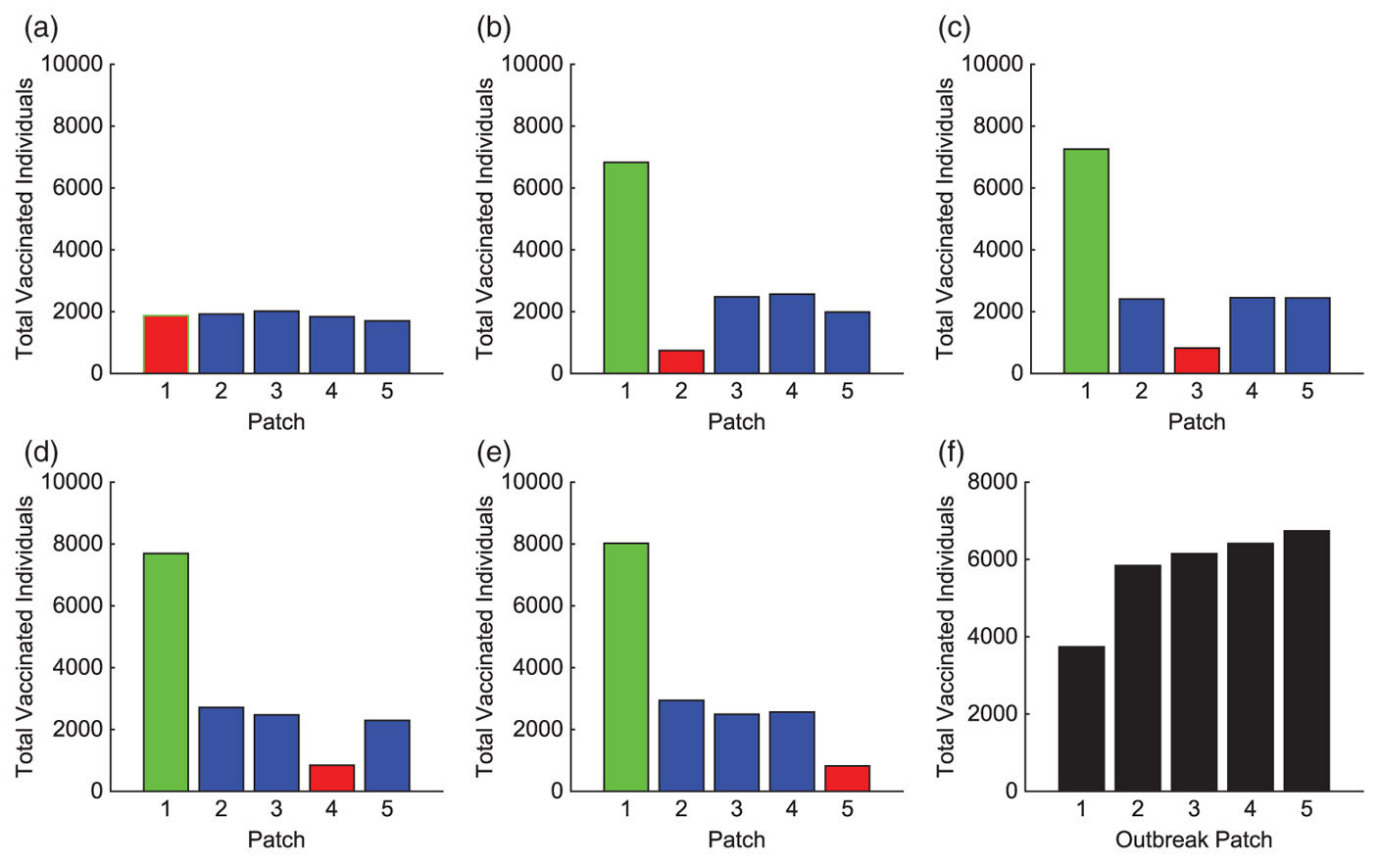
Hub Patch 1 arrangement: total individuals vaccinated in each patch for each scenario. The total vaccinated in metapopulation for each scenario is given in (f). (The hub is shown in green and the outbreak patch is represented by red. When outbreak is in the hub, it is represented by red with green border.)

**Figure 9:**
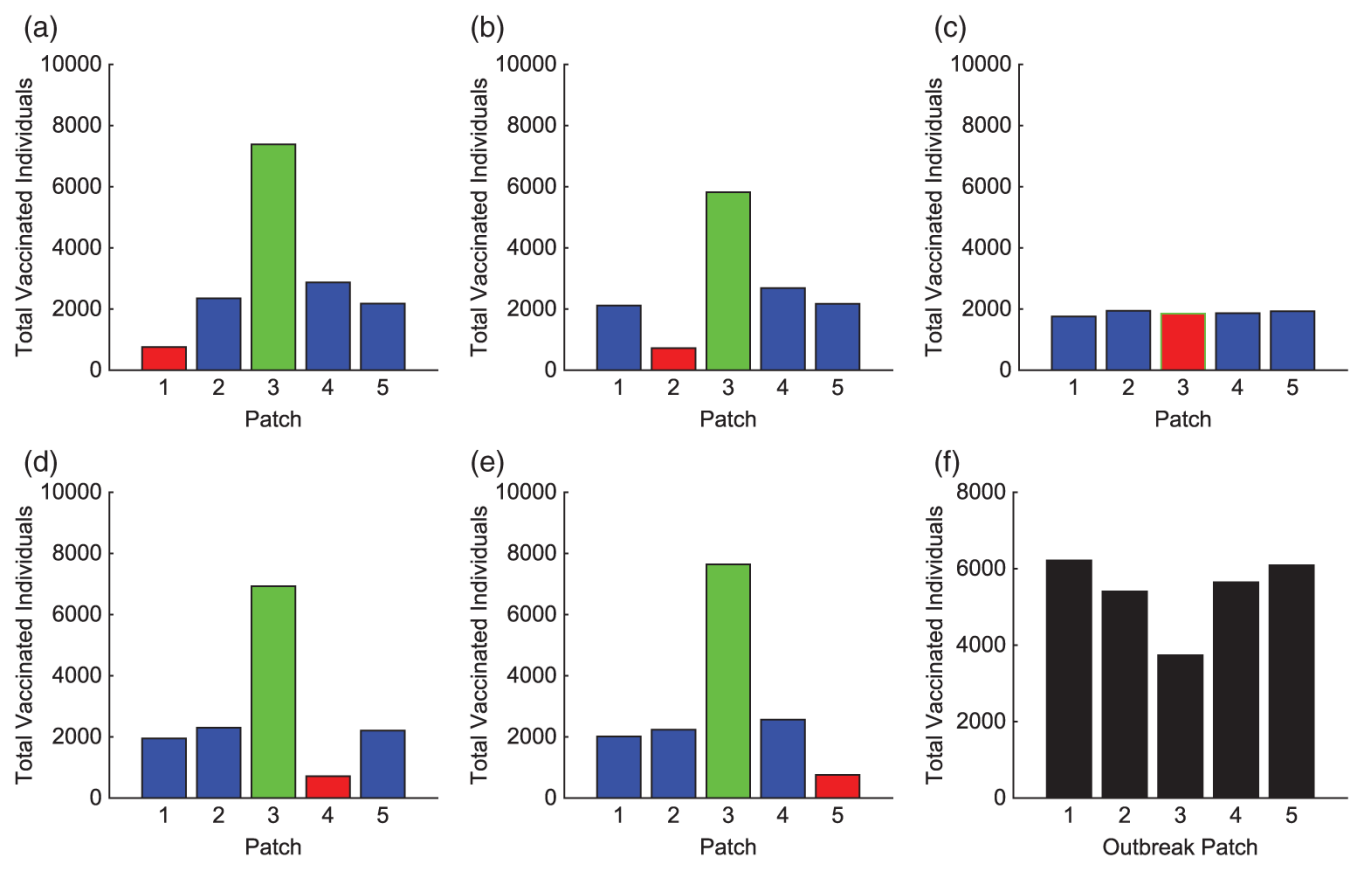
Hub Patch 3 arrangement: total individuals vaccinated in each patch for each scenario. The total vaccinated in metapopulation for each scenario is given in (f). (The hub is shown in green and the outbreak patch is represented by red. When outbreak is in the hub, it is represented by red with green border.)

**Figure 10:**
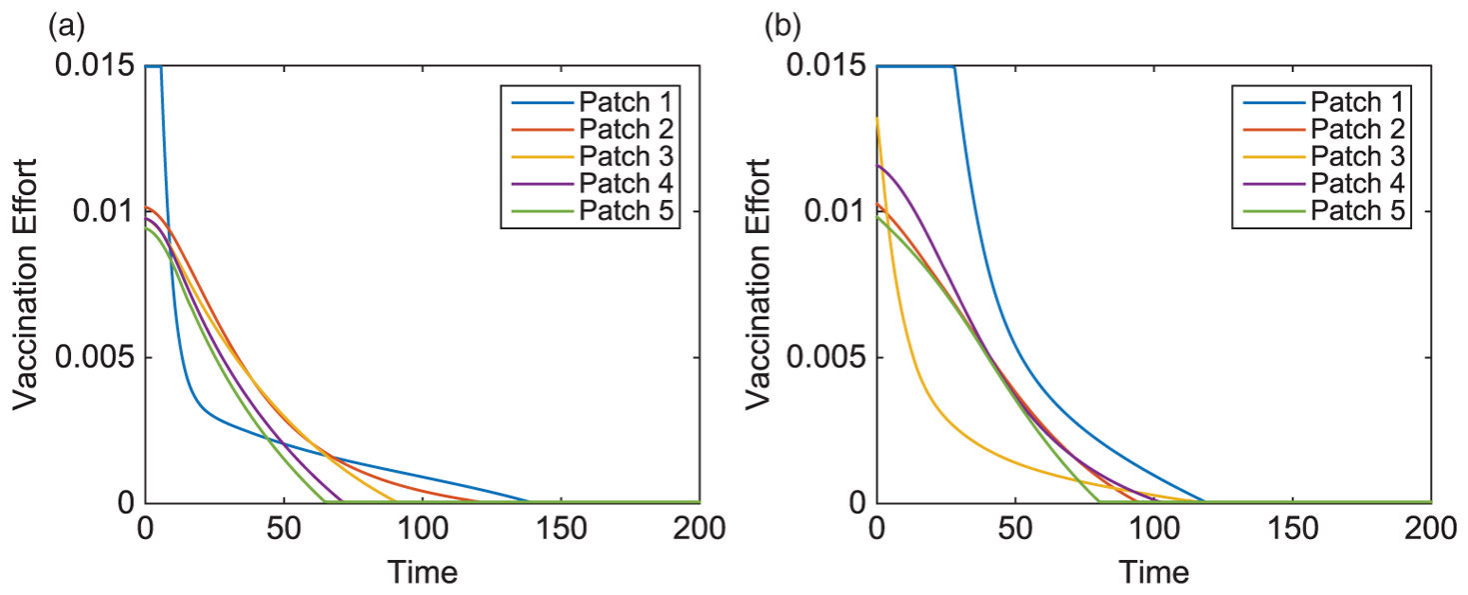
Hub Patch 1 arrangement: vaccination effort of patches with outbreak in hub, Patch 1, and outside in Patch 3.

**Figure 11:**
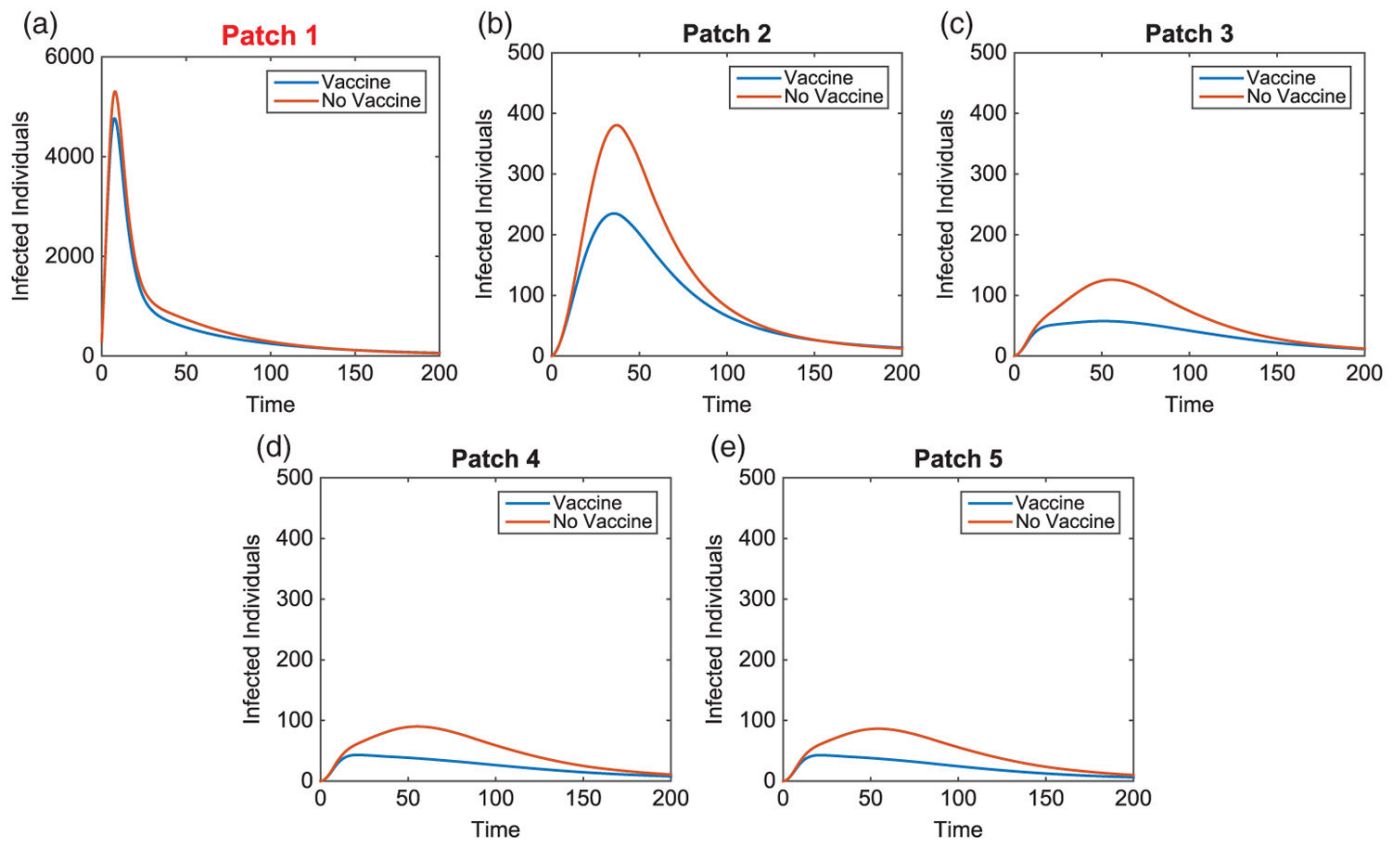
Hub Patch 1 arrangement: infected population dynamics comparison with and without vaccination with outbreak in hub, Patch 1.

We illustrate the example when Patch 1, at the top of river, is the hub. Results when hubs are located in alternative patches of the metapopulation are also discussed (with some illustrations for comparison). Without vaccination, the dynamics of the infected population with outbreaks occurring at the top and middle of the metapopulation are given in Figure 7. For each outbreak scenario, the total infected with and without vaccination is given in Table 6. The total vaccinated in each patch is illustrated in Figure 8. Results for when the outbreak occurs either in the hub patch or outside the hub in Patch 3 are shown in Figures 9–11. There is also discussion when the outbreak occurs in Patch 5.

We draw several conclusions from hub arrangements. First, notice the basic reproduction number of the networks. The location of the hub matters as the highest network **R**_0_ value occurs in the centralized patch.

Regardless of hub location, an outbreak that originates in the hub patch is always hardest to contain due to the difficulty in preventing disease spread to surrounding patches through the hub. Although the total number of infecteds is smallest when an outbreak is in the hub (Table 6), vaccination has the least impact on decreasing the total number of infecteds. Similar results occur with hubs located at other locations in the metapopulation.

**Figure 12:**
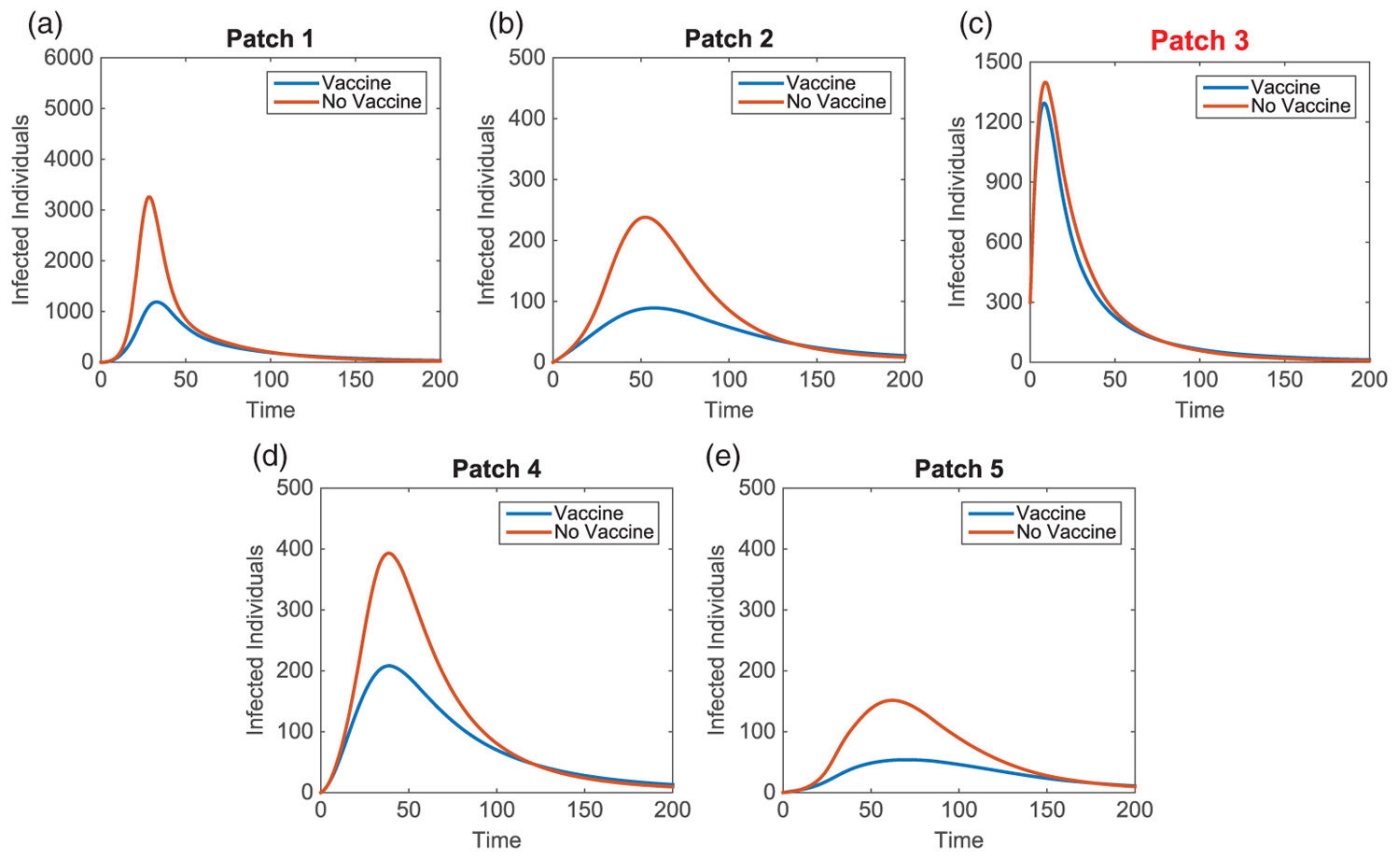
Hub Patch 1 arrangement: infected population dynamics comparison with and without vaccination with outbreak in Patch 3.

The importance of the hub is obvious. With an outbreak outside of the hub, a much different strategy is implemented. In every non-hub outbreak scenario, the number of vaccinations in the hub is significantly higher than in the surrounding patches (see Figures 8 and 12). Maximum effort is sustained longest in the hub and drastically decreases the number of infecteds in the hub. Due to the high connectivities with the hub, outbreaks will spread to the hub quickly and then to the surrounding patches. Using high levels of effort to prevent, or lessen, the spread in the hub in order to limit its spread to the other patches is critical. Limiting the number of infecteds in the hub will ultimately help limit the outbreak in other patches.

Similar to the linear spatial arrangement cases, there are low levels of vaccination administered in the outbreak patch with only a slight decrease in the number of their infecteds. This points to the fact that it is usually too late to contain the outbreak once it has already invaded a patch and more effort should be focused on surrounding patches.

Outside the hub and outbreak patches, the effort spent in other patches follows similar patterns as in the linear arrangement cases. However, there are cases when the strategies differ and the highest number of vaccinations is given to protect patches closer to the hub rather than the outbreak patch. This is seen in Figures 8 and 12. Since any outbreak will almost immediately invade the hub and then progress downstream the network, protecting patches immediately downstream the hub would be important. This is especially true when you take into account the higher movement of pathogen downstream rather than upstream the network.

### 6.3. Linear spatial arrangement with a hotspot

Motivated by the work of Tien *et al.* [30] and Eisenberg *et al.* [14], we consider scenarios with heterogeneity among the patches, where, although population sizes are identical, certain patches possess a higher risk of infectivity. Two potential reasons could be worse sanitation or lower availability of clean water. Areas that have a higher risk of infectivity will be referred to as ‘HSs’. The idea of an HS with accompanying scenarios and the network **R**_0_ is discussed in [30]. We investigate HSs in the linear arrangement.

**Figure 13:**
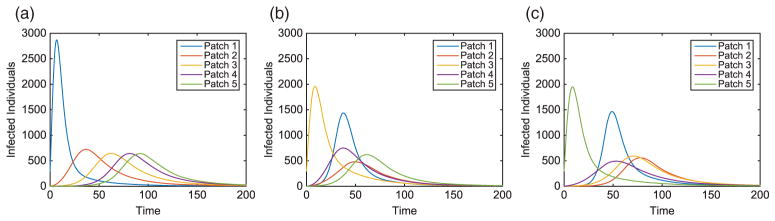
Hot Spot Patch 1 arrangement: infected population dynamics (without vaccination) of a linear arrangement with an HS in Patch 1. The outbreak in the HS (a) is given in comparison with an outbreak in the middle (b) and bottom (c) patches of the metapopulation.

**Figure 14:**
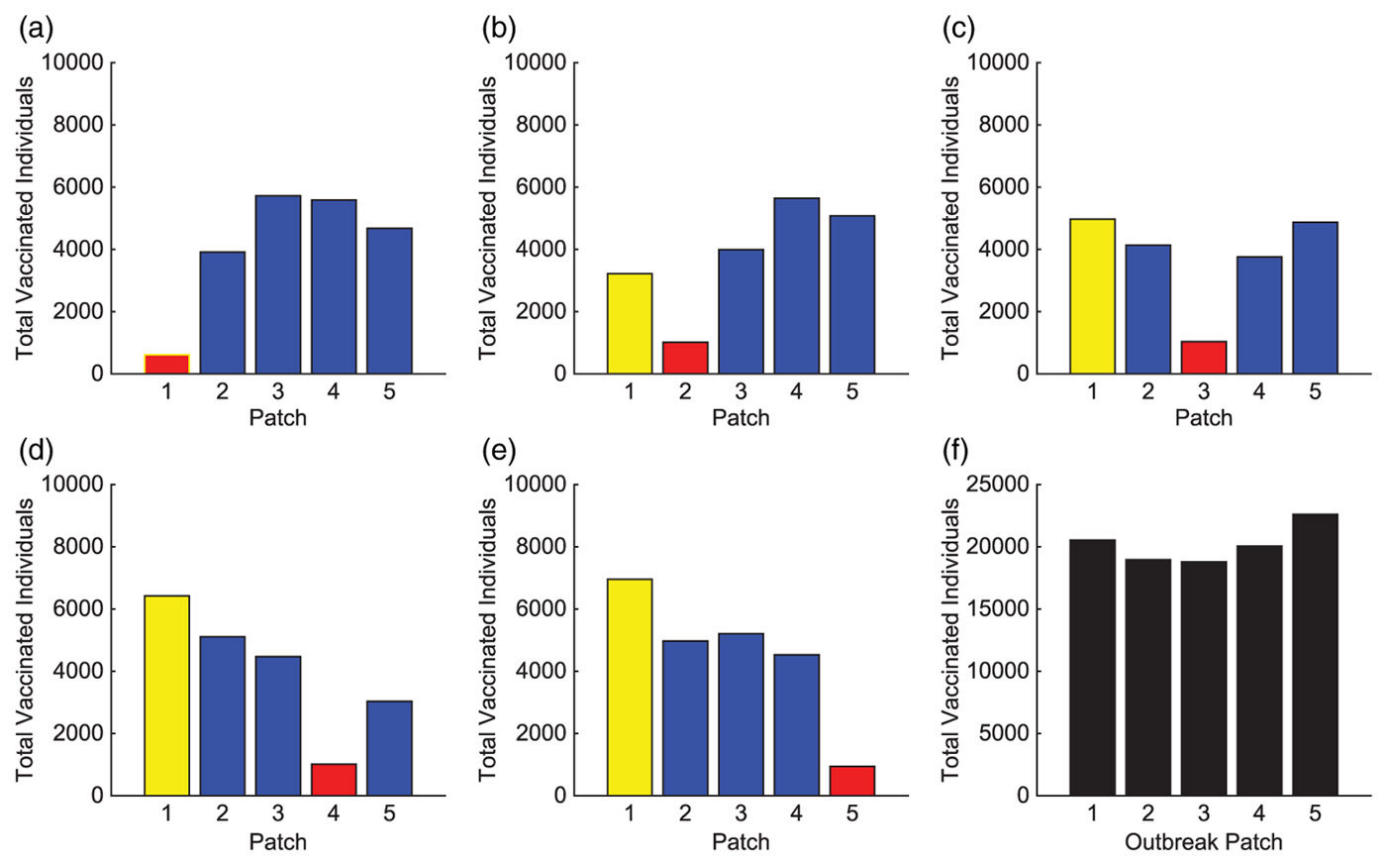
Hot Spot 1 arrangement: total individuals vaccinated in each patch for each scenario. The total vaccinated in metapopulation for each scenario is given in (f). (The HS patch is shown in yellow and the outbreak patch is represented by red. When outbreak is in HS, it is represented by red with yellow border.)

**Figure 15:**
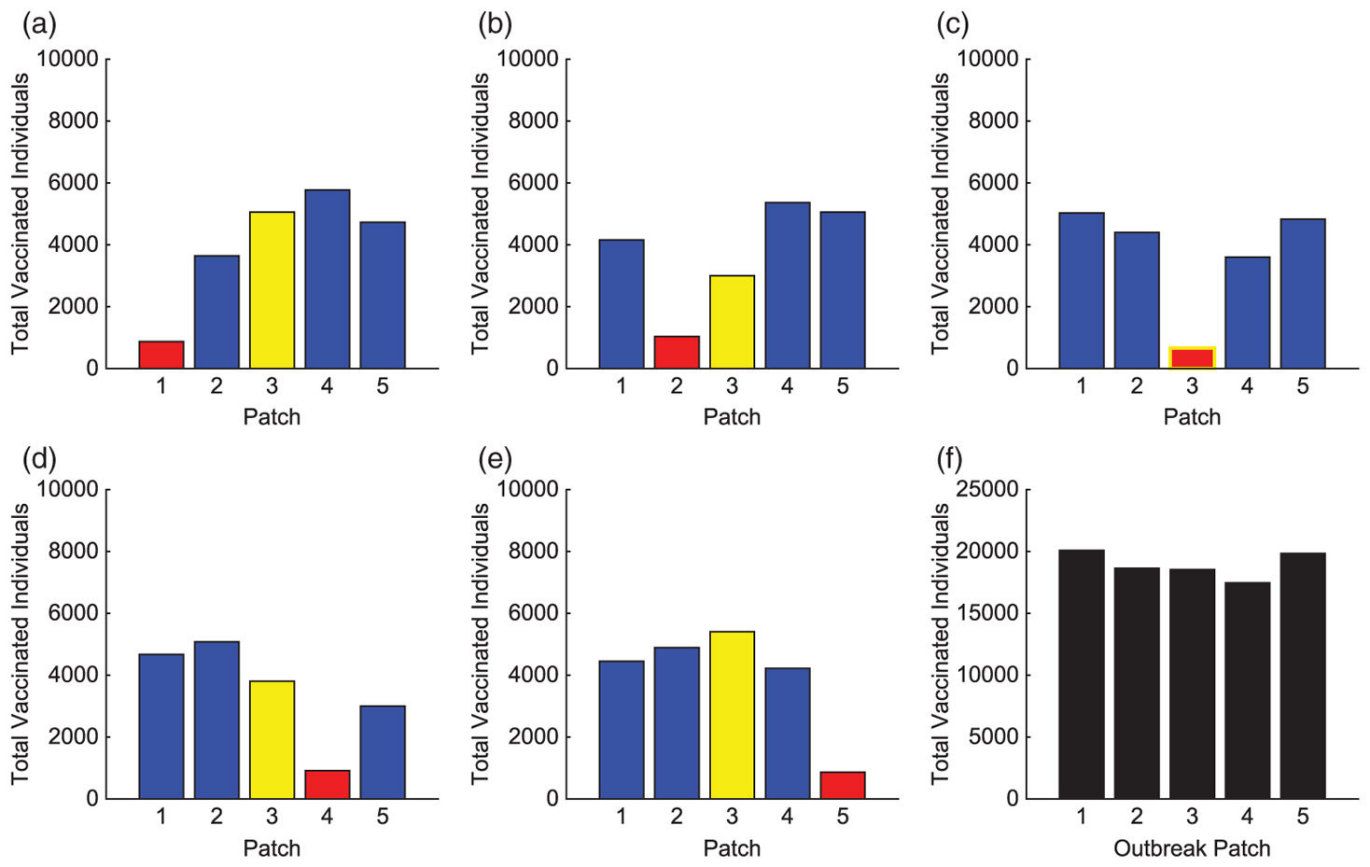
Hot Spot 3 arrangement: total individuals vaccinated in each patch for each scenario. The total vaccinated in metapopulation for each scenario is given in (f). (The HS patch is shown in yellow and the outbreak patch is represented by red. When outbreak is in HS, it is represented by red with yellow border.)

**Figure 16:**
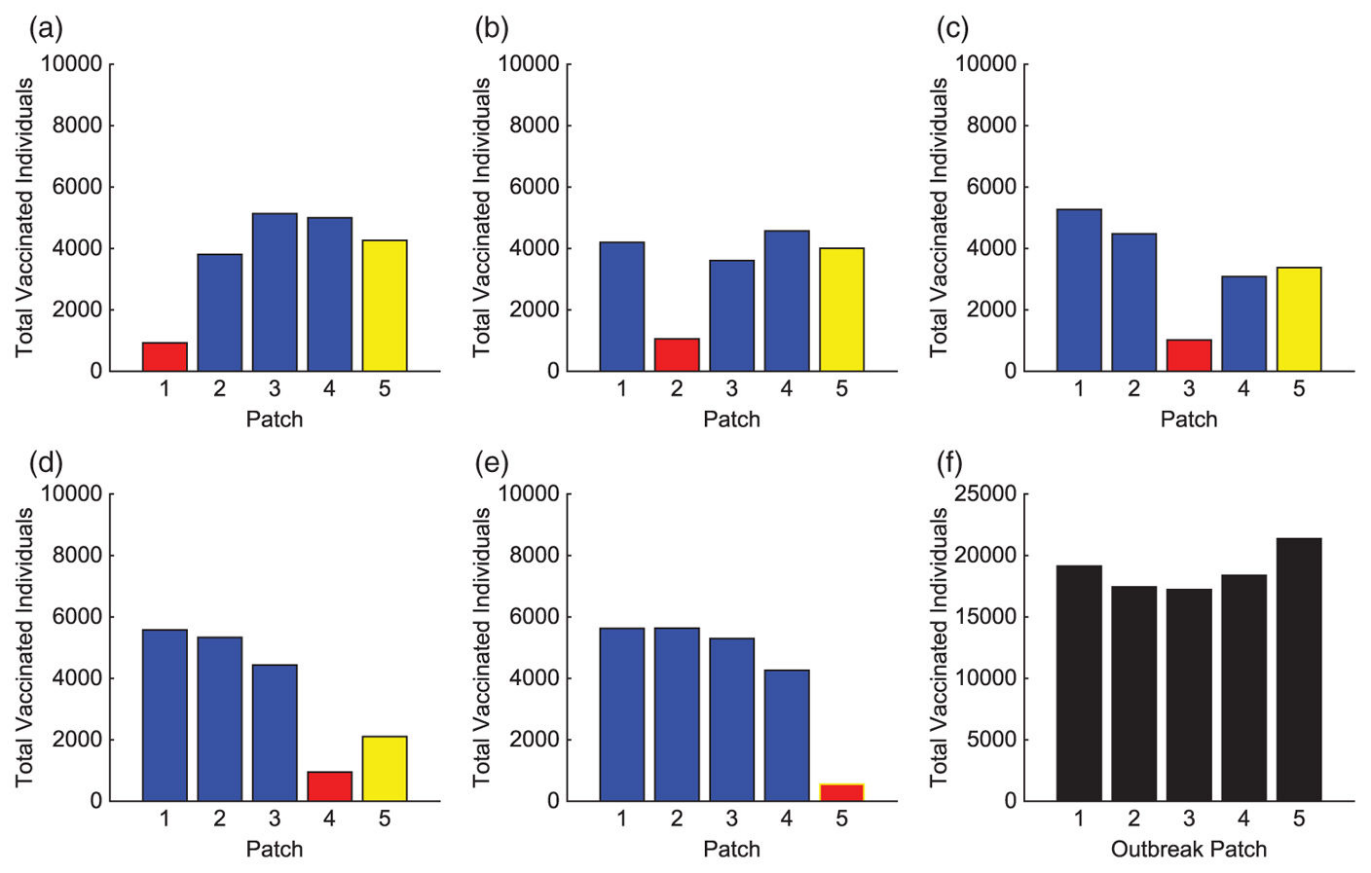
Hot Spot 5 arrangement: total individuals vaccinated in each patch for each scenario. The total vaccinated in metapopulation for each scenario is given in (f). (The HS patch is shown in yellow and the outbreak patch is represented by red. When outbreak is in the HS, it is represented by red with yellow border.)

**Figure 17:**
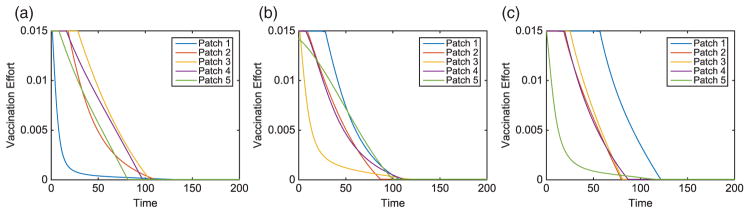
Hot Spot Patch 1 arrangement: vaccination effort for a linear arrangement with an HS in Patch 1, with outbreaks in Patches 1, 3, or 5.

The contact rates for the HS were chosen to be 150% of the other patches, described in Table 7. Similar to the hub, we investigate HSs at varying locations on the metapopulation. We use the same initial conditions as in the previous linear arrangement simulations. These results are compared to scenarios where an HS is located in alternative locations of the metapopulation. The scenario with an HS in Patch 1 is illustrated in Figures 9, 13, and 14. The total infected with and without vaccination when an HS is in Patch 1 is given in Table 9. Due to heterogeneity now incorporated in the network, we illustrate results for HSs in Patches 1, 3, or 5. The total vaccinated in each patch for each scenario are illustrated in Figures 15–17, respectively.

The basic reproduction numbers of the network for varying HS locations are recorded in Table 8. The basic reproduction number in an HS was *R*_0_*HS*__ = 8.84 and $R_{0}^{i}=5.88$ in all surrounding patches.

Tien *et al.* [30] show that for unbalanced linear network arrangements, network risk increases moving downstream, and thus the domain ℛ_0_ increases when HSs are located further downstream. Despite this, the optimal vaccination strategies identified here did not deploy the least control effort in the furthest upstream patch. Similar results occurred within the hub arrangements. This may reflect the fact that implementation of vaccination in our model acts as “damage control” after an outbreak has already begun. Optimal control strategies may differ in the situation of preemptive vaccination.

Another potential reason for the differing results could be our more complicated movement structure, allowing for the movement of the population and the inclusion of hubs in certain cases, not addressed in [30]. Also, our model incorporated pathogen leaving the system both at the upper and lowermost patches in the water arrangement. This eliminates the water compartment in the upper and lowermost patches differing from other patches in the arrangement, in terms of pathogen in the aquatic reservoir. Unlike [30], the best case for an HS, based on network **R**_0_ values, would be in the centre or upstream of the spatial arrangement, but not necessarily at the top.

As seen in our results, disease control efforts are not always highest in patches considered to have the highest network risk, defined as the patch contributing most to the network **R**_0_. Although HSs have higher $R_{0}^{i}$ values, the control effort for each patch varies depending on location and where an outbreak begins (Figures 15–17). This is important to consider when implementing a responsive control strategy.

Regardless of where an outbreak occurs in the metapopulation, we see similar effort levels in the outbreak patch as in previous scenarios. The level of vaccine effort in the HS is dependent on the proximity of an HS to the outbreak patch. The closer the HS is to the outbreak, the less effort is applied there. The farther the distance, the higher the vaccine effort. This is comparable to the hub arrangements with outbreaks outside the hub. There is high effort in nearest neighbour patches but the HS is the priority due to its high potential for invasion. In comparing several results of the linear arrangements, with and without HSs, Figures 3 and 15–17 reveal that the vaccination strategies differ most when the distance between an HS and outbreak is large. When there is no direct connection between the HS and outbreak patch, increasing the effort in the HS almost eradicates the outbreak from this patch.

All simulations incorporated networks with the same magnitude of movement rates, for humans and pathogen. It is important to note that the size of the diffusion parameters governing this movement is important, especially in regard to timing. As human and pathogen movement increase, the speed of outbreak increases. Vaccinations are then distributed on a shorter time scale with the initial effort in each patch increasing. However, the timing and prioritization of patches are maintained.

## 7. Conclusions and discussion

After developing and analyzing an optimal control problem for a waterborne disease metapopulation model, we used numerical simulations to approximate solutions. We considered three arrangements and found the optimal vaccination strategies. We investigated a linear spatial arrangement with strictly nearest neighbour movement, hub spatial arrangements with one patch having a larger population, higher connectivity, and higher movement rates, and linear spatial arrangements with patches of higher infectivity known as ‘HSs’. We sought answers to the question of where control efforts should be focused depending on metapopulation structure and path dynamics.

We give several guiding strategies based on our spatial arrangement scenarios below:

### The outbreak patch almost always receives least amount of effort

Once the disease invades a patch, it becomes too difficult to contain in that patch so effort is spent elsewhere. This result differs in cases where the outbreak patch is the hub or a centrally located HS. In these cases, the effort is almost evenly distributed among the system.

### Due to the directional flow of rivers affecting pathogen movement, more vaccinations can be administered when outbreaks occur downstream rather than upstream

Pathogen moving more rapidly downstream than up allows for fewer individuals to be vaccinated. Slower upstream movement allows more time to vaccinate before the pathogen arrives upstream, thus increasing the amount of protection. The role of water pathways and the ability of the pathogen to spread along river networks must be considered when implementing a vaccination program. Effort is distributed depending on the location of populations in relation to the outbreak.

### Outbreaks are hardest to contain when starting in a hub, which implies that preemptive prevention of disease invasions in hubs is highly important

The added connectivity of the hub makes the outbreak much harder to contain due to disease invasion in every patch of the metapopulation shortly after outbreak begins. When the outbreak occurs within a hub, vaccination effort is spent evenly among surrounding patches but its effect in preventing the disease is minimal.

### With outbreaks beginning outside of a hub, the focus of vaccinations should be in the hub

There is greater importance in protecting the hub due to its potential to spread the outbreak to all patches of the metapopulation.

### Location of high risk areas (‘HSs’) in relation to the outbreak matters

Despite the existence of HSs in the network, if these areas are not located in close proximity to the outbreak location, it is not necessarily more important to contain outbreak in the HS than in surrounding patches (location matters). Depending on location, it may be more important to vaccinate nearest neighbours instead to prevent the outbreak from ever reaching the HS.

Our work shows convincingly that spatial arrangements and heterogeneity in features (such as HSs, sizes, and connectivity) are important to management strategies. These features can have a signinicant impact on intervention decisions. An investigation of more heterogeneous sets of patches will be important to further understand spatial dynamics for disease spread and intervention strategies. These optimal control tools, applied to the varying scenarios, can give guiding ‘rule of thumb’ strategies for the management of disease epidemics when concerned about spatial features of a landscape.

Results are shown for response control strategies once an outbreak has already occurred. They provide the optimal way to control an outbreak but not necessarily how to eradicate outbreak completely. There is a need to find preemptive control strategies for a spatial landscape, whether before an outbreak occurs or in an endemic setting. There is question on how much vaccination would be needed to completely eliminate the outbreak from the population. Work by Tien, Eisenberg, and their collaborators investigated this while investigating the role of spatial features on the disease risk of networks [14, 30]. Our work used a maximum vaccination effort consistent with the recently implemented vaccination program in Haiti. It would be important to consider what vaccination effort would be necessary to get ahead of the outbreak to control it and consider the time frame of control and whether shorter time frames with higher doses would alter the control strategies.

## Figures and Tables

**Table 1:** Description of compartments with units for metapopulation model.

Compartments	Description	Units
*S_i_*	Susceptible individual density	ind. km^−2^
*I_i_*	Infected individual density	ind. km^−2^
*R_i_*	Immune due to vaccination or recovery	ind. km^−2^
*N_i_*	Total population density	ind. km^−2^
*W_i_*	Scaled pathogen concentration in water reservoir	ind. km^−2^

**Table 2:** Description of parameters with units for metapopulation model.

Parameters	Description	Units
*μ_i_*	Birth and death (non-disease related)	day^−1^
$\beta_{I}^{i}$	Person–person contact rate	km^2^ ind.^−1^ day^−1^
$\beta_{W}^{i}$	Reservoir–person contact rate	km^2^ ind.^−1^ day^−1^
*γ_i_*	Duration of infectiousness of the disease	day^−1^
*δ_i_*	Death due to disease	day^−1^
*ξ_i_*	Pathogen decay in water	day^−1^
*d_S_*	Diffusion coefficient (for all susceptibles)	day^−1^
*d_R_*	Diffusion coefficient (for all recovered)	day^−1^
*d_I_*	Diffusion coefficient (for infected)	day^−1^
*d_W_*	Diffusion coefficient (for pathogen)	day^−1^

**Table 3:** Parameter values for numerical simulations with identical patches.

Parameters	Description	Value
*μ_i_*	Birth and death (non-disease related)	1.00E–4
$\beta_{I}^{i}$	Person–person contact rate	2.64E–5
$\beta_{W}^{i}$	Reservoir–person contact rate	1.21E–4
*γ_i_*	Duration of infectiousness of the disease	0.25
*δ_i_*	Death due to disease	5.0E–4
*ξ_i_*	Mean survival of pathogen in water	7.56E–3
*d_S_*	Diffusion coefficient (for all susceptibles)	5.00E–3
*d_R_*	Diffusion coefficient (for all recovered)	5.00E–3
*d_I_*	Diffusion coefficient (for infecteds)	1.00E–3
*d_W_*	Diffusion coefficient (for pathogen)	3.00E–5
*ρ*_d_	Coefficient for pathogen leaving network downstream	3.00E–5
*ρ*_u_	Coefficient for pathogen leaving network upstream	3.00E–6
v_max_	Max vaccination	0.015
*T*	Final time (days)	200
*A_i_*	Cost to minimize infecteds	1
*B_i_*	Quadratic cost of vaccination	300,000
*C_i_*	Linear cost of susceptibles vaccinated	3.25

**Table 4:** Linear arrangement: total number of infected individuals in metapopulation, with and without vaccine.

Outbreak	Total infecteds (no vaccine)	Total infecteds (with vaccine)	Difference (%)
Patch 1	49,173	21,694	55.88
Patch 2	49,192	25,353	48.46
Patch 3	49,190	25,755	47.64
Patch 4	49,003	23,447	52.15
Patch 5	48,646	17,579	63.86

**Table 5:** Basic reproduction number of the network for varying hub patch locations.

Hub	Patch 1	Patch 3	Patch 5
Network **R**_0_	7.92	8.01	7.92

**Table 6:** Hub Patch 1 arrangement: total number of infected individuals in metapopulation, with and without vaccine.

Outbreak	Total infecteds (no vaccine)	Total infecteds (with vaccine)	Difference (%)
Patch 1	48,493	38,716	20.16
Patch 2	48,712	33,134	31.98
Patch 3	49,396	33,188	32.81
Patch 4	49,413	32,325	34.58
Patch 5	48,782	30,357	37.77

**Table 7:** Parameter values for infectivity in HS and surrounding patches.

Parameter	Description	Value in HS	Value elsewhere
*β_I_*	Person–person contact rate	3.96E^−5^	2.64E^−5^
*β_W_*	Reservoir–person contact rate	1.82E^−4^	1.21E^−4^

**Table 8:** Basic reproduction number for the network with varying HS patches.

Hotspot	Patch 1	Patch 3	Patch 5
Network **R**_0_	6.72	6.81	6.73

**Table 9:** Hot Spot Patch 1: total number of infected individuals in metapopulation, with and without vaccine.

Outbreak	Total infecteds (no vaccine)	Total infecteds (with vaccine)	Difference (%)
Patch 1	49,223	22,384	54.53
Patch 2	49,569	27,475	44.57
Patch 3	49,668	29,082	41.45
Patch 4	49,555	25,943	47.65
Patch 5	49,232	19,702	59.98
